# Corrigendum to “Evaluation of Some Mechanical Properties of a Maxillofacial Silicon Elastomer Reinforced with Polyester Powder”

**DOI:** 10.1155/2020/7187159

**Published:** 2020-09-28

**Authors:** Yagthan Mohammed Haider, Zainab Salih Abdullah, Abdalbseet A. Fatalla, Ghasak H. Jani, Norehan Mokhtar

**Affiliations:** ^1^Department of Prosthodontics, College of Dentistry, University of Baghdad, Baghdad, Iraq; ^2^Advanced Medical and Dental Institute, University Science Malaysia, George Town, Malaysia

In the article titled “Evaluation of Some Mechanical Properties of a Maxillofacial Silicon Elastomer Reinforced with Polyester Powder” [1], Dr. Abdalbseet A. Fatalla was missing from the authors' list. Dr. Fatalla shared his knowledge and experience during the study design and manuscript preparation and also provided the polyester particles used in the research. The corrected authors' list is shown above.
